# Evaluation on curative effects of combined acupuncture plus physical therapy for treating idiopathic facial paralysis: A protocol for a systematic review and meta-analysis

**DOI:** 10.1097/MD.0000000000023121

**Published:** 2020-11-13

**Authors:** Cui-Yi Zhang, Yan Huang, Ke Zhang, Fang Dong

**Affiliations:** a Department of Neurology, Shenzhen Hospital of Guangzhou University of Chinese Medicine (Futian), Shenzhen; b Department of neurology, Guangdong Provincial Hospital of Chinese Medicine, The Second Affiliated Hospital of Guangzhou University of Chinese Medicine, Guangzhou; c Department of traditional Chinese medicine, Shenzhen Far East Maternity Hospital; d Medical Records Statistics Division, Shenzhen Hospital of Guangzhou University of Chinese Medicine (Futian), Shenzhen, Guangdong, China.

**Keywords:** acupuncture, efficacy, idiopathic facial paralysis, protocol, systematic review

## Abstract

**Background:**

The present study primarily aims to evaluate how effective acupuncture combined with physical therapy for the treatment of idiopathic facial paralysis.

**Methods:**

The PubMed database was searched (1946 to September 2020), the EMBASE data were also searched (January 1946 to September 2020), moreover, the Cochrane Central Register of Controlled Trials was searched (all years), and finally, the China National Knowledge Infrastructure (CNKI) was also included in the searching of electronic databases. The searching of publications did not include any language constraints. The titles and abstracts were scrutinized by a pair of authors to identify relevant studies. The efficacy of the association in the combination of acupuncture and physical therapy as a method of treatment for idiopathic facial paralysis was evaluated according to the pooled risk ratio (RR), mean differences (MD), or standardized mean difference (SMD) with the corresponding 95% confidence intervals (95% CI). A pair of authors conducted an autonomous risk assessment of the bias that would be introduced when the Cochrane Risk of Bias Tool is used. A pair of authors autonomously extracted data with the aid of a customized data extraction form. The RevMan 5.3 statistical analysis software was utilized for conducting the statistical analysis.

**Results:**

The final results will be presented in a scientific journal that will be peer-reviewed.

**Conclusion:**

It is expected that the proposed systematic review and meta-analysis of acupuncture combined with physical therapy for treating idiopathic facial paralysis will provide reliable evidence for clinical application.

**OSF registration number:**

DOI 10.17605/OSF.IO/RPCSE (https://osf.io/rpcse/)

## 1 Introduction

Idiopathic facial paralysis is also acknowledged as Bell's palsy. It is an acute peripheral facial neuropathy. It is a commonly occurring condition which disfigures a patient's facial appearance. It results in the paralysis of structures that are innervated by the facial nerve.^[1,2]^ The common clinical symptoms of idiopathic facial paralysis include facial weakness, sudden onset, auricular pain, dysgeusia, unilateral paralysis, headache, dry eye, epiphora, and xerophthalmia.^[3,4]^ It has been estimated that the annual prevalence rate of facial paralysis is between 20 and 30 cases per 100,000 adults. The prevalence rate between males and females is quite similar.^[2,5]^ As a result, therapeutic methods to overcome this condition have gained the attention of researchers and clinical practitioners.

Admittedly, the etiology of idiopathic facial paralysis is yet to be conclusively established. However, recent studies have provided evidence which indicates that idiopathic facial paralysis is primarily caused by the reactivation of latent herpes simplex virus type 1 (HSV type1).^[3,6,7]^ Besides the main cause, other conditions such as, meningiomas, polyneuropathic, traumatic, HIV infection, Lyme disease, and inflammatory disease also cause facial paralysis.^[8–10]^ In general, the treatment of idiopathic facial paralysis involves corticosteroids, antiviral drugs, vitamin B, physiotherapy, acupuncture, surgery, among others.^[11–16]^

Acupuncture is regarded as one of the most ancient form of traditional therapy. Historically, acupuncture has been utilized as a therapeutic strategy for many diseases, such as idiopathic facial paralysis. Acupuncture is renowned as a practical and cost-effective therapeutic strategy for treating numerous diseases with little side effects. Physical therapeutic interventions such as, facial exercises, electrotherapy, endurance, biofeedback, and massages have been used to treat facial paralysis. The goal of these modalities is to improve muscle and nerve functionalities through exercise or electrotherapy.

Research and data corresponding to the utilization of acupuncture for treating idiopathic facial paralysis are quite sparse; the reported results are contradicting with each other, and lack conclusiveness. The goal of the present study is to conduct a comprehensive systematic review using studies that have already been published. The systematic review will assess the effectiveness of using combined acupuncture plus physical therapy for the treatment of idiopathic facial paralysis.

## 2 Methods

The present systematic review has been listed and included in the Open Science Framework (OSF, https://osf.io/). This systematic review is registered under the DOI number of the 10.17605/OSF.IO/RPCSE. The development of this protocol was in line with the Preferred Reporting Items for Systematic Review and Meta-Analyses guidelines for protocols (PRISMA-P).^[17]^

## 3 Eligibility criteria

### 3.1 Types of studies

This review includes all the randomized or quasi-randomized (alternate or other systematic allocation) controlled trials that have studied any form of acupuncture therapy compared with no treatment, placebo treatment, drug treatment, or other therapeutic interventions.

### 3.2 Types of participants

Participants are patients who have been diagnosed with idiopathic facial paralysis. There is no age, sex, or ethnical constraints; hence, participants of all ages from any ethnicity who are males and females with varying levels of severity are included in this systematic review. Healthy individuals or patients diagnosed with other identified causes were excluded.

### 3.3 Types of interventions and comparisons

This review included past studies that have investigated any form of acupuncture therapy compared with no treatment, placebo treatment, drug treatment, or other therapeutic interventions. Acupuncture therapy referring to needling or the combination of needling and moxibustion were considered.

### 3.4 Types of outcome measures

#### 3.4.1 Primary outcomes

Primary outcomes were: paralysis score; physical facial disability indexs and social facial disability index; and recovery rate of supracordal/infracordal lesions.

#### 3.4.2 Second outcomes

Secondary outcomes were:

1. The existence of motor synkinesis, contracture, hyperkinesia, facial spasm, or crocodile tears preferably 6 months following the onset.
2. Incomplete recovery after a year.
3. Serious effects attributable to intervention such as pain or an aggravated condition.
4. The consideration of additional outcomes will depend on review findings; expected outcomes include accompanying promotion of mental health.

## 4 Search methods

### 4.1 Electronic searches

Four electronic databases were searched, namely, PubMed (1946 to September 2020), the EMBASE (January 1946 to September 2020), the Cochrane Central Register of Controlled Trials (all years), as well as the China National Knowledge Infrastructure (CNKI). As mentioned before, there were not any constraints on the language of the published articles.

### 4.2 Searching other resources

1. The reference lists in each of the identified studies.
2. The first authors of each of the unpublished trials included in the review will be contacted to obtain additional insights.
3. To source additional studies, ClinicalTrials.gov (www.ClinicalTrials.gov), Google scholar, and all primary studies were searched.

### 4.3 Search strategy

To obtain the optimum feasibility and specificity of the review, a customized search strategy is implemented. The strategy emphasizes on Medical Subject Headings (MeSH), such as “acupuncture therapy” or “acupuncture treatment” and “controlled clinical trials,” as well as text searches of keywords, such as “facial palsy" and “facial paralysis” (an alternate term adopted in certain instances to reference a similar construct). The strategy implemented to search the PubMed database is provided in Table 1.

**Table 1:** The Search Strategy for the PubMed Database.

Number	Items
#1	“Bell Palsy”[Mesh]
#2	“Bell Palsy”[Title/Abstract]
#3	“Palsies, Bell”[Title/Abstract]
#4	“Palsy, Bell”[Title/Abstract]
#5	“Facial Paralysis, Idiopathic”[Title/Abstract]
#6	“Facial Paralyses, Idiopathic”[Title/Abstract]
#7	“Idiopathic Facial Paralyses”[Title/Abstract]
#8	“Idiopathic Facial Paralysis”[Title/Abstract]
#9	#1 OR #2 OR #3 OR #4 OR #5 OR #6 OR #7 OR #8
#10	“Randomized Controlled Trial” [Publication Type]
#11	“controlled clinical trial” [Publication Type]
#12	“randomised” [Title/Abstract]
#13	“randomized” [Title/Abstract]
#14	#11 OR # 12 OR #13
#15	Acupuncture[Mesh]
#16	“Acupuncture”[Title/Abstract]
#17	#15 OR #16
#18	#9 AND #14 AND #17

## 5 Data collection and analysis

### 5.1 Selection of studies

**Figure 1:**
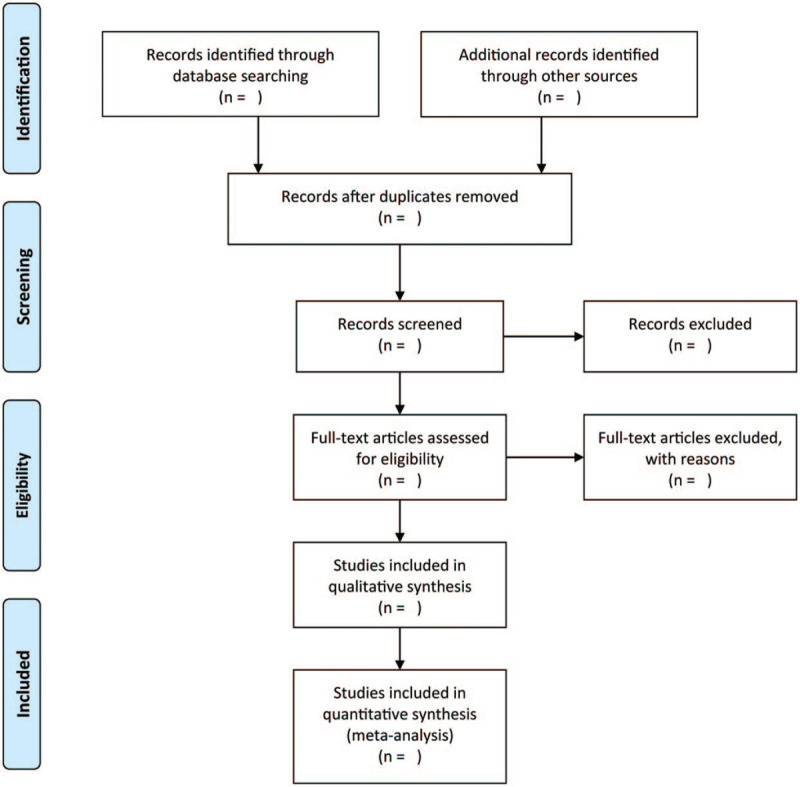
Flow diagram of the literature search.

The titles and abstracts of the studies that were identified through the search were independently scrutinized by tw2 authors. Full texts were successfully obtained for all the potentially relevant studies that were selected for an autonomously carried out assessment. The pair of authors determined the trials that satisfied the inclusion criteria. Any disagreements regarding the selection of studies were resolved by consensus. The flow diagram of the literature search was shown in Figure 1.

### 5.2 Data extraction and management

Data with regards to the participants, methods, interventions, outcomes, and conclusions were extracted autonomously by 2 authors. A customized data extraction form was used to achieve this. Wherever applicable, missing data will be obtained from the trial authors. All disagreements regarding data extraction were resolved through consensus. The reference management system in EndNote X9 will be utilized to manage the studies. Duplication will also be facilitated by EndNote X9.

### 5.3 Assessment of risk of bias in included studies

The Cochrane Collaboration's risk tool was utilized to perform an autonomous evaluation of the methodological quality of the studies that were selected by the 2 authors. Disagreements between the 2 authors shall be resolved through discussion or by consulting a third independent author. The new “Risk of bias” assessments and the “Risk of bias” tables, described in the Cochrane Handbook is also included in the updated version, these cover 7 items.^[18]^ Each criterion was scored as either “high risk,” “low risk,” or “unclear risk.”

### 5.4 Measures of treatment effect

The risk ratio (RR) with 95% confidence intervals (CI) based on the fixed-effects model or random-effects model was calculated for dichotomous data. Meanwhile, the mean difference (MD) or standardized mean difference (SMD) with 95% CI based on the fixed-effects model or random-effects model was calculated for continuous outcomes.

### 5.5 Assessment of heterogeneity

The heterogeneity will be evaluated by the *χ*^*2*^ test, and it was assumed to be present when the significance level was lower than 0.10.^[19–21]^ During the presence of significant heterogeneity, attempts were made to explicate the key differences based on the clinical features of the studies included in the review.

### 5.6 Assessment of reporting biases

In the case wherein the number of included studies exceed 10, a funnel plot will be utilized for assessing report bias. Statistical investigation will be fulfilled by Egger test.^[22,23]^

### 5.7 Assessment of sensitivity analysis

A sensitivity analysis was conducted, which neglected trials that involved participants with contrasting clinical features, or trials with substandard methodological quality.

## 6 Ethics and dissemination

The results of this review will be published in a peer-reviewed journal. It is not necessary to obtain an ethical approval since the personal data of patients are not used.

## 7 Discussion

Idiopathic facial paralysis has many adverse effects on the daily life and life standard of an individual. It causes pain and changes the appearance of individuals. In most cases, treatment with corticosteroid is not required. Acupuncture is renowned as a noninvasive external physiotherapy; it has been widely used for treating idiopathic facial palsy. However, the present status of using acupuncture combined with physical therapy as a form of therapy for individuals diagnosed with idiopathic facial paralysis lacks conclusive evidence of its efficacy and safety. This review provides compelling evidence and guidance to improve future clinical practices.

## Author contributions

ZCY, HY, and DF conceptualized the study. ZCY wrote the first draft of the protocol, and all authors participated in further drafting. ZCY, ZK, and HY revised the qualitative methods and interview guide. All authors read and approved the final manuscript.

**Conceptualization:** Cuiyi Zhang.

**Data curation:** Cuiyi Zhang.

**Formal analysis:** Cuiyi Zhang.

**Funding acquisition:** Yan Huang.

**Methodology:** Yan Huang.

**Project administration:** Yan Huang, Ke Zhang, Fang Dong.

**Resources:** Ke Zhang, Fang Dong.

**Software:** Ke Zhang, Fang Dong.

**Supervision:** Ke Zhang.

**Validation:** Cuiyi Zhang, Fang Dong.
